# Negative moods correlate with craving in female methamphetamine users enrolled in compulsory detoxification

**DOI:** 10.1186/1747-597X-7-44

**Published:** 2012-10-30

**Authors:** Wenwen Shen, Yu Liu, Longhui Li, Yisheng Zhang, Wenhua Zhou

**Affiliations:** 1 Ningbo Addiction Research and Treatment Center, School of Medicine, Ningbo University, 42 Xibei Street, Ningbo, Zhejiang 315010, P.R.China; 2 Department of Obstetrics, Ningbo First Hospital, 59 Liuting Street, Ningbo, Zhejiang 315010, P.R.China

**Keywords:** Methamphetamine, Addiction, Craving, Mood distress, Detoxification

## Abstract

**Background:**

Methamphetamine (METH) use, especially in females, has become a growing public health concern in China. In this study, we aimed to characterize the factors that contributed to drug craving in female METH users under isolated compulsory detoxification. We characterized factors contributing to craving such as duration of detoxification, history of drug use and self-reported mood state.

**Methods:**

Subjects (N=113) undergoing a 1- to 3-year METH detoxification program were recruited from the Zhejiang Compulsory Detoxification Center for Women. The Questionnaire of METH-use Urge (QMU) was used to evaluate the level of craving for METH. The Abbreviate Profile of Mood States (A-POMS) was applied as an assessment for the negative mood disturbances.

**Results:**

The participants were at a mean age of 25.2, primarily lowly educated and unemployed, and single. Smoking was the only route of METH administration at an average dose of 0.5 g/day, and 4 times/week. The reported craving level was positively correlated with the negative mood disturbances and the weekly dose of METH, but independent of the duration of detoxification. Furthermore, all five aspects of negative mood disturbances, including fatigue, bewilderment, anxiety, depression and hostility, were shown to positively correlate to the self-reported craving level after controlling for weekly dose of METH.

**Conclusions:**

The data demonstrate a robust correlation between mood distress and craving for METH. Our results call for close evaluation of mood distress in treatment of METH users in China.

## Background

Amphetamine-type stimulant (ATS) use is rapidly increasing worldwide. It is estimated that 3.4–20.7 million people have used ATS in East and South-East Asia, which is approximately 1.4% of the population aged 15–64 years [1]. The number of methamphetamine (METH) users has increased dramatically in China in recent years [2]. In 2001, there were approximately 160,000 registered ATS users ^a^ , which consisted of 17.3% of the total registered illicit drug addicts in China [3]. However, by the end of 2010, the number of registered ATS users had increased to approximately 430,000, which consisted of 31.0% of all registered drug users [4].

The evidences have shown that the females can sometimes have worse outcomes than the males in relation to METH use. For example, one study has suggested that women are more likely to become dependent on METH than men [5]. Similar vulnerability to METH has been implicated in several species of rodents. For instance, female Wistar rats are more vulnerable to the acquisition of METH self-administration [6]. Meanwhile, female C57BL/6J mice exhibit a more potent METH-induced conditioned place preference than the male mice, and these gender differences may be facilitated by estradiol [7]. A number of studies have also shown that female METH users exhibit more severe psychiatric symptoms [8-11]. Researchers in Taiwan have found that 43.4% of female METH users suffer from psychiatric problems, 35.9% demonstrate suicidal behavior, and 11.0% are diagnosed with mood disorders [8]. In contrast, only about 31.1% men have psychiatric problems, and 5.6% and 8.9% of them are diagnosed with suicidal behavior and mood disorders, respectively [8]. Similarly, one study in San Diego have shown the high prevalence of depressive symptoms among female METH users, with severe depressive symptoms observed in 60% of them [9] A study focused on adolescents has also found the more symptoms in female METH users by Symptom Checklist-90R, with enhanced cortisol secretion following a social stressor [12]. Taken together, probably vulnerability to METH use in women suggests the necessity for a focus on the female METH users, with attention to the mood states.

In China, drug addicts with confirmed relapses are required to receive isolated compulsory detoxification for 1–3 years [13], where the confirmed relapse means registered drug users being caught abusing drugs repeatedly more than twice. In Zhejiang province, approximately 8.0% of registered drug users were considered as relapsed users, and were enrolled in the detoxification program. During the program, participants were isolated from the communities for at least one year, and evaluated by behavioral improvement. Usually, they receive physical training for the first three months. And in the following months, they get paid work in the working days, and receive education on the weekends regarding drug control law, virtue, and occupational skills. Individuals with severe depressive symptoms or stress are able to receive personal psychological counseling. However, not any forms of cognitive behavioral therapies have yet been introduced into the system. The effect of such compulsory treatment has not been fully evaluated among METH users.

Drug craving is a critical component of addiction, and serves to elicit relapse in psychostimulant users [14,15]. Current data show that craving for METH decreases within two months after abstinence [16]. However, the longer-term effect of abstinence on craving for METH has not been studied. The high rate of relapse suggests that craving might persist or reemerge after a long period of abstinence. Moreover, mood distress is known to increase the craving for nicotine [17-21], alcohol [22,23] and heroin [24] in drug abstinence subjects. However, it is not known whether mood distress also increases the craving for METH.

The purpose of the present study was to determine the factors that contributed to METH craving in the women under isolated compulsory detoxification, who might be expected to have more salient mood problems than their male partners. We focused on the time-dependent effect of the detoxification program and self-reported mood states in female METH users We hypothesized that craving might recur after a longer period of detoxification, and it might be affected by negative moods states.

## Methods

### Participants

The investigation protocols have been approved by the Institutional Review Board of the Ningbo Addiction Research and Treatment Center according to the Declaration of Helsinki (No. 2010–01). Female METH users, who only or primarily used METH in the last year prior to enroll, were recruited from the Compulsory Detoxification Center for Women in Zhejiang Province. During the detoxification program, subjects were isolated from the community, and spot checks of the urine tests were done every three months to ensure the abstinence. No positive urine test for any of the illicit drugs (e.g. morphine, amphetamine or cocaine) was found in the center during the past two years before our investigation.

Criteria of inclusion were meeting the diagnosis of METH abuse or dependence by DSM-IV, while subjects with diagnosis of abuse or dependence of other substances were excluded. The diagnosis of METH dependence or abuse was made upon self-reports of METH use during the last month when participants had free access to the drug. Voluntary participation was required and subjects received stationery gifts, upon request, for participating. Personal information collected from participants was kept confidential, and the personal data collected were used only for this study. The interviews were undertaken under a separated room, and the officers of the detoxification center were not allowed to stand by or listen to the interviews. Participants gave written informed consent after a full explanation of the study had been provided, and each subject completed the survey under the direction of the trained researchers. This investigation was conducted in Mandarin, and took approximately 30 minutes for each participant to complete.

### Questionnaires

A battery of questionnaires was designed and discussed by the researchers. One month before the investigation, researchers discussed the questionnaires item-by-item to exclude any ambiguity that might be introduced in the investigation. Investigators were also taught to express with more understandable oral language which the drug users commonly used. For most of the items, choices were provided to facilitate the investigation, expected for the questions that needed numerical answers. Subjects were allowed to choose multiple-choices answers or to provide their own answers.

Demographic variables included age, ethnicity, education level, occupation, marital status and children. Characteristics of METH use included the onset age of METH use, total duration of METH use (years), frequency (times/week), dose (gram/day), route of administration, environment of METH use in the 12 months prior to incarceration, and the duration of present detoxification.

The Questionnaire of METH-use Urge (QMU) was modified from the Questionnaire of Smoking Urge-brief [25] by replacing the word “smoking” with “using METH”. The Chinese version of Questionnaire of Smoking Urge-brief had been used in a previous study on nicotine craving [26]. The QMU contains 10 items. Participants were asked to indicate how strongly they currently agreed or disagreed with each item using a Likert scale from 1 (strongly disagree) to 7 (strongly agree). The participant responses were classified by two factors. Factor 1 addresses the “intention/desire to use METH” (items 1 and 6), and factor 2 (items 4, 8, and 9) addresses the “anticipation of relief of negative effect/urgent desire to use METH”. It is moderately to highly correlated with the Visual Analog Scale for METH Craving (VASC) (coefficient for QMU factor 1 and VASC: 0.78; for factor 2 and VASC: 0.69, for total scores and VASC: 0.77).

The Abbreviate Profile of Mood States (A-POMS) [27] used in this study is a revision of an earlier version of the Profile of Mood States, and has been modified and translated into Chinese [28]. It contains 40 items rated on a 5-point scale from 0 (not at all) to 4 (extremely). Subscales include 5 negative mood factors (tension-anxiety, depression, anger-hostility, fatigue, and confusion-bewilderment) and 2 positive mood factors (vigor-activity and self-esteem). Total mood disturbance (TMD) was calculated as the sum of negative mood factors minus the positive mood factors, vigor and self-esteem. Higher scores of negative moods indicate a greater degree of mood disturbance. These data are based on mood states experienced and reported ‘during the past week, including today’. The POMS is a well-developed, highly reliable method to assess mood disturbance [29]. It is moderately to highly correlated with other scales, including the Visual Analog Mood Scales, the State-Trait Anxiety Inventory, and the Beck Depression Inventory, with coefficients ranging from 0.72 to 0.79 [30].

### Data analyses

The data were tested for normality using the Shapiro-Wilk method and had a skewed distribution. Therefore, the data were analyzed by two-tailed Mann–Whitney U-test and Kruskal-Wallis H test. Categorical variables were analyzed by two-tailed chi-square or Fisher’s exact test. A Spearman correlation was used to determine the relationship between craving, mood, characteristics of drug use, and participant demographics. A Spearman partial correlation was used to investigate the independent factors related to METH craving. The data were analyzed using SPSS for Windows (version 16.0, SPSS, Chicago, IL, USA), and significance was set at *p* < 0.05.

## Results

### General information

A total of 113 females were included in this study. Participant demographics and characteristics of METH use are reported in Table 1. Every participant self-administered METH via smoking. Self-report of multiple substances of abuse were present in 40.7% (45/112) of subjects. Other types of ATSs, such as Magu, a methamphetamine-caffeine mixture, were used by 38.9% of the participants, and Ecstasy was used by 12.4%. The top three frequently used non-ATSs were ketamine (28.6%), cannabis (15.2%), and heroin (8.9%).

**Table 1:** **Participant Demographics and Characteristics of Meth Use** (**N**=**113**)

**Demographic factors**	**Mean ± SD/Number (percentage)**
Age (years)	25.2 ± 6.9
Education Illiterate	3 (2.7%)
Primary school	34 (30.1%)
Middle school	60 (53.1%)
High school /tech school	14 (12.4%)
College	2 (1.8%)
Occupation Unemployed	58 (55.2%)
Employed	28 (26.7%)
Self-employed	19 (18.1%)
Marital status Not married	90 (80.4%)
Married	11 (9.8%)
Divorced	11 (9.8%)
Children None	91 (81.3%)
One	20 (17.9%)
More than one	1 (0.9%)
**Characteristics of METH use**	
Age beginning meth usage	22.7 ± 6.7
Years of meth use	2.0 ± 1.4
Dose (g/day)	0.6 ± 0.6
Frequency (times/week)	4.8 ± 4.1
Duration of detoxification (months)	8.7 ± 4.8

Based on the criteria of DSM-IV, 101 participants met the diagnosis of METH dependence, and 12 were METH abusers. Subjects with METH dependence had longer durations (z = 2.312, *p* < 0.05), more frequent use patterns (z=3.742, *p* <0.001), and larger daily doses (z = 2.730, *p* <0.01) of METH use, and higher scores of QMU factor 2 (z = 1.976, *p* < 0.05). No significant differences of QMU factor 1 (z=1.848 *p*=0.065), QMU total scores (z=1.541, *p*=0.123), and negative mood states (TMD) (z=1.537, *p*=0.124) were found between the two groups.

**Figure 1:**
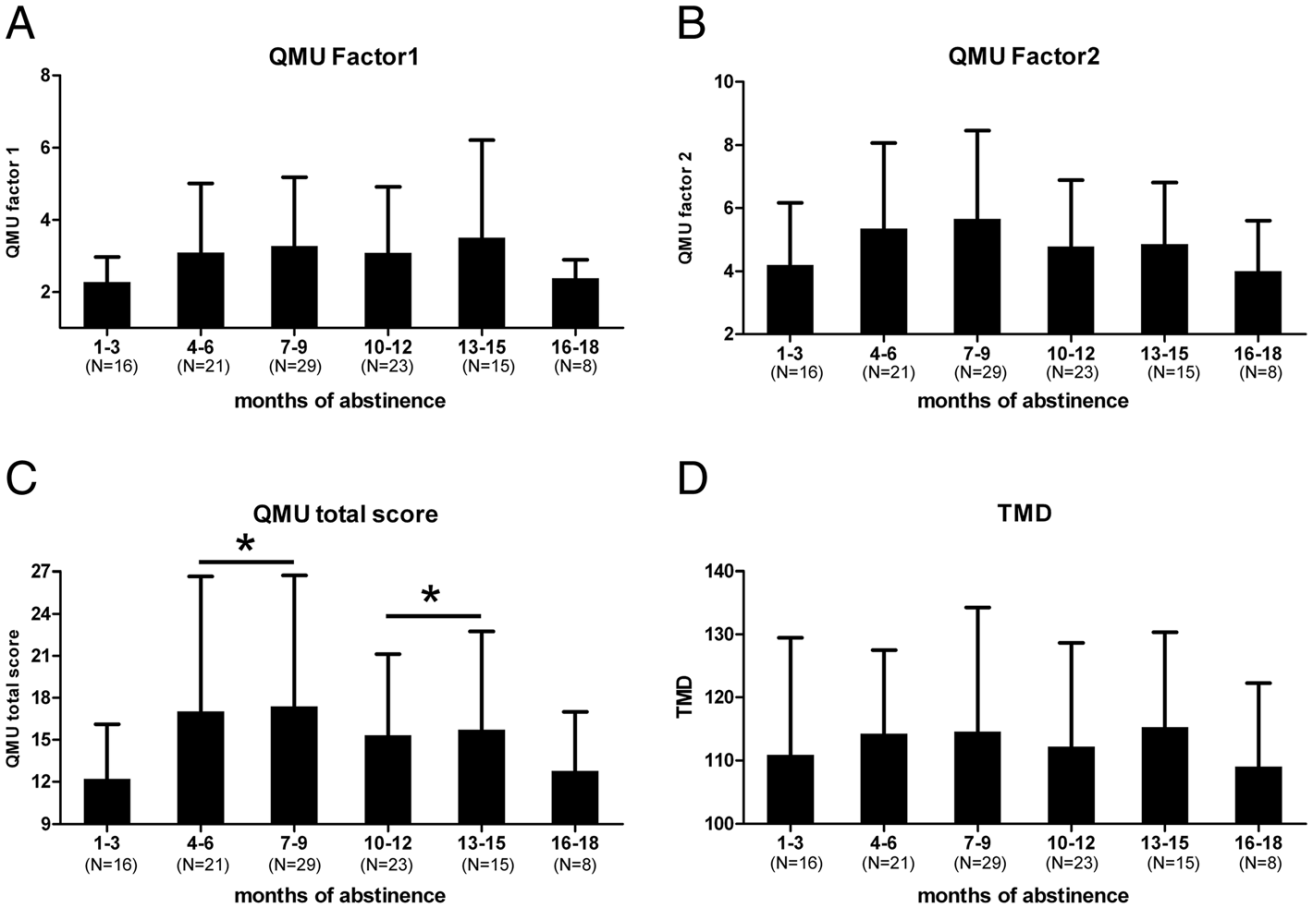
**The distribution of QMU scores and negative mood states in the participants following the different durations of abstinence.** QMU, Questionnaire of METH-use Urge. Factor 1 addresses “intention/desire to use meth” and factor 2 addresses “anticipation of relief of negative effect/urgent desire to use meth”. TMD: Total Mood Disturbance. *: QMU total scores were higher in subjects who were abstinent for 4–9 months and 10–15 months, compared to those are abstinent for 1–3 months (Kruskal Wallis tests, *p*<0.05).

As shown in Figure 1, the craving level for METH and the TMD in 3-month intervals according to the duration that each individual had been enrolled in the program. After combining the neighboring groups with comparable craving levels, Kruskal Wallis tests were performed in subjects at detoxification durations of 1–3 months, 4–9 months, 10–15 months, and more than 15 months. It was found that the QMU total scores were higher in subjects who were abstinent for 4–9 months and 10–15 months (Chi-square =9.26, *p* <0.05). However, no significant difference was observed in the QMU factor 1, 2 and TMD.

### Mood distress and former drug use independently affect craving level

**Figure 2:**
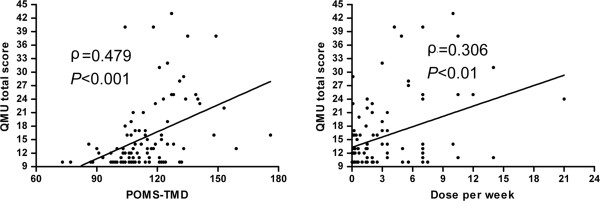
**Craving levels were positively correlated to total mood disturbance** (**left**) **and the weekly dose of former METH use (right).** POMS, Profile of Mood State. TMD, Total Mood Disturbance. QMU, Questionnaire of METH-use Urge.

Factors related to the increased craving were screened by Spearman coefficient analysis. As shown in Figure 2, significant correlations were found between QMU factors and negative mood states, as well as the weekly METH dose. Other characteristics of the participants, such as age, education level, marital status, work experience, the age of first METH use, and the duration of METH use were not significantly correlated with the current craving level. TMD can be decomposed into five negative mood ingredients including fatigue, bewilderment, anxiety, depression and hostility, minus two positive mood factors of vigor-activity and self-esteem. Statistical analysis showed that all the five negative mood factors, but not the two positive mood factors, were closely related to the QMU score (Table 2).

**Table 2:** **The Spearman correlation coefficients between ratings of POMS and scores of Questionnaires of METH**-**use Urge** (**QMU**)

	**Spearman coefficients**		
**A**-**POMS**	QMU factor 1	QMU factor 2	QMU total score
Fatigue	0.275**	0.330**	0.341***
Confusion-bewilderment	0.236*	0.332**	0.342***
Tension-anxiety	0.291**	0.382***	0.346***
Depression	0.263**	0.444***	0.391***
Anger-hostility	0.277**	0.402***	0.404***
Vigor-activity	−0.028	−0.065	−0.050
Self-esteem	−0.046	−0.033	−0.025
Total mood disturbance (TMD)	0.355***	0.503***	0.479***

*p< 0.05, **p< 0.01, ***p< 0.001.

Partial correlation analysis (Table 3) was used to determine the independent factors that related to the increased QMU scores. After controlling for overall mood distress, the weekly dose of METH use was significantly related to the total score of QMU and QMU factor 2, but not QMU factor 1. TMD had a significant correlation with QMU scores after controlling for weekly dose, duration of METH dose, or duration of detoxification. Moreover, each of the five negative mood factors including fatigue, bewilderment, anxiety, depression and hostility, were related to all QMU scores (total score of QMU, QMU factor 1 and 2) after controlling for weekly dose of meth.

**Table 3:** **The Spearman Partial Correlations with Questionnaires of METH**-**use Urge** (**QMU**) **Scores**

	**Spearman partial coefficients**		
Models	QMU Factor 1	QMU Factor 2	QMU Total score
1: Dose per week controlled for TMD	0.177	0.236*	0.261*
2. Fatigue controlled for weekly dose	0.260*	0.314**	0.326**
3. Confusion controlled for weekly dose	0.214*	0.310**	0.320**
4. Tension-anxiety controlled for weekly dose	0.268**	0.359***	0.320**
5. Depression controlled for weekly dose	0.245*	0.432***	0.376***
6. Anger-hostility controlled for weekly dose	0.273**	0.405***	0.408***
7. TMD controlled for dose of meth	0.331**	0.481***	0.455***
8. TMD controlled for duration of meth use	0.330**	0.493***	0.467***
9. TMD controlled for duration of detoxification	0.353***	0.504***	0.477***
10. 7+8	0.314**	0.477***	0.450***
11. 7+8+9	0.314**	0.479***	0.450***

*p< 0.05, **p< 0.01, ***p< 0.001.

## Discussion

The primary finding of present study was that the craving ratings increased, but the ratings of total mood disturbance did not change following the abstinence in the female METH users. The data demonstrated the impact of mood distress on craving level in female METH users. All of the five ingredients of negative moods, including hostility, depression, anxiety, bewilderment, and fatigue, showed an positive relationship to increased craving level. The weekly dose of METH use was the only one among historical factors that related to METH craving, and the effect of higher-dose METH use history on craving was primarily independent of the present negative mood states.

The present data were consistent with the previous studies that mood distress increases the craving for nicotine [17-21], alcohol [22,23] and heroin [24] in drug abstinence subjects. METH abusers who also have mood problems are more vulnerable to psychiatric diseases. For example, it has been shown that relapse of METH psychosis can be evoked by stress [31,32]. In addition, major depression and suicidal ideation is commonly observed in patients seeking treatment, especially in females [11]. Proper psychosocial therapy can not only lower the degree of craving and drug use [33,34], but also help to prevent relapse and exacerbation of psychiatric diseases [35,36]. The ultimate goal of compulsory detoxification is to prevent relapse among METH users who will be released in 1–3 years. Our results suggest that negative mood distress should be carefully evaluated during the mandatory treatment. Therefore, providing the suitable treatment for the mood disorders should be a key component of the compulsory detoxification program.

Although some literature suggests that craving for METH may greatly decreases within the first two months of abstinence [16], our study implicated a persistence of desire to use the drug, which might be easily promoted by mood disturbance. A progressive elevation of cue-induced craving over an extended period of abstinence was first suggested in 1986 in cocaine users [37]. This phenomenon was lately named as incubation of drug craving, and repeatedly confirmed in rodents with different substances of abuse [38]. In fact, the present data showed the more craving in the female METH users who exposed to the higher doses of METH. Therefore, acquiring a full history of METH use for each individual would be necessary to anticipate the increased METH craving for women enrolled in treatment.

Biological basis of the long lasting craving has been indicated in many studies. For instance, imaging studies have shown reduced dopamine transporter density and reduced dopamine D2 receptors in the stratum [39,40], and reduced glutamate in prefrontal gray matter [41] in abstinent psychostimulant users and non-human primate. These persistent corticolimblic dysregulation can cause increased baseline levels of stress [42] and deficits in inhibitory control [43]. And drug-seeking behavior recurs once drug cue or stressful environment is presented. Meanwhile, the long- term craving has been suggested as a result of the formation of GluA2-lacking AMPARs in the nucleus accumbens [38]. The incubation of craving has also been linked with the increased neuronal activity in the ventral medial prefrontal cortex, nucleus accumbens core, and central amygdale [38]. Taken together, the persistent craving may result from the neural deficits caused by the drugs, as well as neural adaption to the drugs. The detailed mechanisms need to be clarified in further studies.

Environmental context of incarceration may also contribute to the elevated craving. Studies have shown that rodents exhibit enhanced incubation of craving when put in an isolated environment [44] or with the no access of exercise [45]. Similarly, mandatory treatment with subjects isolated from the community may confront the same problem as the experimental animals. Thus, the efficacy of the treatment might be weakened when subjects lived in the relatively close environment. Although the total ratings of mood distress of female METH users did not change following the abstinence in the isolated detoxification Center, introduction of the more activities in a more open environment for female METH users is therefore suggested.

As in all studies, this data should be carefully interpreted. This is a cross-sectional study. Therefore, the data generated from subjects with different duration of compulsory detoxification program may not necessarily represent the time course of mood changes and cravings in individuals in the program. Moreover, our data cannot be applied to describe the time courses of cravings in women who are not in the compulsory detoxification program but remain abstinent from METH. Future longitudinal studies are necessary to understand the course of chronic withdrawal symptoms for each patient as well as patient outcomes once they return to their communities. Additionally, the data collected on the characteristics of drug use, mood state, and craving level are based on participant self-reports, but because patients were guaranteed confidentiality, we concluded that the data are reliable and valid.

## Conclusions

This study is the first to demonstrate a correlation between mood distress and craving for METH. It is also the first study that focuses on the isolated compulsory detoxification program in China. Our results call for appropriate psychosocial therapies to offer coping skills and to alleviate mood disorders in women with METH dependence in detoxification treatment.

## Endnotes

^a.^ In P.R.China, individuals with records of using illicit drugs in the local police station are registered in the Narcotics Control Bureau of the Ministry of Public Security, and they are called registered drug users. After registration, they are asked to report to the local police office regularly, and underwent the urine test for illicit drugs randomly. If the records of drug use for the individual were clean for three years, then the individual is considered as recovered and is removed from the registration list. The list helps the government to understand the prevalence of drug use, as well as to treat the drug users.

## Abbreviations

A-POMS: The Abbreviate Profile of Mood States; ATS: Amphetamine-type stimulant; METH: Methamphetamine; QMU: Questionnaire of Meth-use Urge-belief; TMD: Total mood disturbance.

## Competing interests

The authors declare that they have no competing interests.

## Authors’ contributions

WS participated in the interviewing, recorded the data, performed the data analysis, and drafted the manuscript. YL designed the questionnaires, participated in the interviewing, and revised the manuscript. LL helped to design to questionnaires, and participated in interviewing. YZ designed to questionnaires and participated in interviewing. WZ conceived of the study, participated in the coordination and made the final manuscript. All authors read and approved the final manuscript.

## References

[B1] United Nations Office on Drugs and Crime World Drug Report 2010 2010 Vienna: United Nations Publications

[B2] Liu Z Cao J Lu X Mu Y Lian Z Epidemiological study of central stimulants and other related psychoactive substance abuse Chin J Drug Depend 2002 11 286 293

[B3] Office of the National Narcotics Control Commission Annual report on drug control in China 2001 Beijing: Ministry of Public Security of China

[B4] Office of the National Narcotics Control Commission Annual report on drug control in China 2011 Beijing: Ministry of Public Security of China

[B5] Brecht ML O’Brien A von Mayrhauser C Anglin MD Methamphetamine use behaviors and gender differences Addict Behav 2004 29 89 106 10.1016/S0306-4603(03)00082-0 14667423

[B6] Roth ME Carroll ME Sex differences in the acquisition of IV methamphetamine self-administration and subsequent maintenance under a progressive ratio schedule in rats Psychopharmacology (Berl) 2004 172 443 449 10.1007/s00213-003-1670-0 14654996

[B7] Chen HH Yang YK Yeh TL Cherng CF Hsu HC Hsiao SY Yu L Methamphetamine-induced conditioned place preference is facilitated by estradiol pretreatment in female mice Chin J Physiol 2003 46 169 174 15074837

[B8] Lin SK Ball D Hsiao CC Chiang YL Ree SC Chen CK Psychiatric comorbidity and gender differences of persons incarcerated for methamphetamine abuse in Taiwan Psychiatry Clin Neurosci 2004 58 206 212 10.1111/j.1440-1819.2003.01218.x 15009828

[B9] Semple SJ Zians J Strathdee SA Patterson TL Psychosocial and behavioral correlates of depressed mood among female methamphetamine users J Psychoactive Drugs 2007 Suppl 4 353 366 18284102 10.1080/02791072.2007.10399897

[B10] King G Alicata D Cloak C Chang L Psychiatric Symptoms and HPA Axis Function in Adolescent Methamphetamine Users J Neuroimmune Pharmacol 2010 5 582 91 10.1007/s11481-010-9206-y 20358305 PMC2974768

[B11] Darke S Torok M McKetin R Kaye S Ross J Patterns of psychological distress related to regular methamphetamine and opioid use Addiction Res Theor 2011 19 121 127 10.3109/16066351003695631

[B12] King G Alicata D Cloak C Chang L Psychiatric symptoms and HPA axis function in adolescent methamphetamine users J Neuroimmune Pharmacol 2010 5 582 591 10.1007/s11481-010-9206-y 20358305 PMC2974768

[B13] Liu Y Liang J Zhao C Zhou W Looking for a solution for drug addiction in China: exploring the challenges and opportunities in the way of China’s new Drug Control Law Int J Drug Policy 2010 21 149 154 10.1016/j.drugpo.2009.10.002 19896818

[B14] Rohsenow DJ Martin RA Eaton CA Monti PM Cocaine craving as a predictor of treatment attrition and outcomes after residential treatment for cocaine dependence J Stud Alcohol Drugs 2007 68 641 648 17690796 10.15288/jsad.2007.68.641

[B15] Galloway GP Singleton EG How long does craving predict use of methamphetamine? Assessment of use one to seven weeks after the assessment of craving: Craving and ongoing methamphetamine use Subst Abuse 2009 1 63 79 19898674 PMC2773437

[B16] 3rd Urschel HC Hanselka LL Gromov I White L Baron M Open-label study of a proprietary treatment program targeting type A gamma-aminobutyric acid receptor dysregulation in methamphetamine dependence Mayo Clin Proc 2007 82 1170 1178 10.4065/82.10.1170 17908523

[B17] Cordovil De Sousa Uva M Luminet O Cortesi M Constant E Derely M De Timary P Distinct effects of protracted withdrawal on affect, craving, selective attention and executive functions among alcohol-dependent patients Alcohol Alcohol 2010 45 241 246 20207627 10.1093/alcalc/agq012

[B18] Fucito LM Juliano LM Depression moderates smoking behavior in response to a sad mood induction Psychol Addict Behav 2009 23 546 551 19769439 10.1037/a0016529 PMC2749968

[B19] Carter BL Lam CY Robinson JD Paris MM Waters AJ Wetter DW Cinciripini PM Real-time craving and mood assessments before and after smoking Nicotine Tob Res 2008 10 1165 1169 10.1080/14622200802163084 18629726 PMC4346280

[B20] Blalock JA Robinson JD Wetter DW Schreindorfer LS Cinciripini PM Nicotine withdrawal in smokers with current depressive disorders undergoing intensive smoking cessation treatment Psychol Addict Behav 2008 22 122 128 18298238 10.1037/0893-164X.22.1.122

[B21] al’Absi M Carr SB Bongard S Anger and psychobiological changes during smoking abstinence and in response to acute stress: prediction of smoking relapse Int J Psychophysiol 2007 66 109 115 10.1016/j.ijpsycho.2007.03.016 17544533 PMC2443944

[B22] Sinha R Fox HC Hong KA Bergquist K Bhagwagar Z Siedlarz KM Enhanced negative emotion and alcohol craving, and altered physiological responses following stress and cue exposure in alcohol dependent individuals Neuropsychopharmacology 2009 34 1198 1208 10.1038/npp.2008.78 18563062 PMC2734452

[B23] de Castro V Fong T Rosenthal RJ Tavares H A comparison of craving and emotional states between pathological gamblers and alcoholics Addict Behav 2007 32 1555 1564 10.1016/j.addbeh.2006.11.014 17174480

[B24] Epstein DH Willner-Reid J Vahabzadeh M Mezghanni M Lin JL Preston KL Real-time electronic diary reports of cue exposure and mood in the hours before cocaine and heroin craving and use Arch Gen Psychiatry 2009 66 88 94 10.1001/archgenpsychiatry.2008.509 19124692 PMC2943840

[B25] Toll BA Katulak NA McKee SA Investigating the factor structure of the Questionnaire on Smoking Urges-Brief (QSU-Brief) Addict Behav 2006 31 1231 1239 10.1016/j.addbeh.2005.09.008 16226843 PMC2527734

[B26] Liu Y Sun HQ Bao YP Li SX Beveridge TJ Di XL Yang FD Lu L Subjective, cognitive/psychomotor, and physiological effects of aripiprazole in Chinese light and heavy smokers Drug Alcohol Depend 2009 101 42 52 10.1016/j.drugalcdep.2008.10.024 19070440

[B27] Grove JR Prapavessie H Preliminary evidence for the reliability and validity of an abbreviated Profile of Mood States Int J Sport Psychol 1992 23 93 109

[B28] Zhu BL Brief introduction of POMS scale and its model for China Journal of Tianjin University of Sport 1995 10 35 37

[B29] Cranford JA Shrout PE Iida M Rafaeli E Yip T Bolger N A procedure for evaluating sensitivity to within-person change: can mood measures in diary studies detect change reliably? Pers Soc Psychol Bull 2006 32 917 929 10.1177/0146167206287721 16738025 PMC2414486

[B30] Nyenhuis DL Yamamoto C Luchetta T Terrien A Parmentier A Adult and geriatric normative data and validation of the profile of mood states J Clin Psychol 1999 55 79 86 10.1002/(SICI)1097-4679(199901)55:1<79::AID-JCLP8>3.0.CO;2-7 10100834

[B31] Yui K Goto K Ikemoto S Nishijima K Yoshino T Ishiguro T Susceptibility to subsequent episodes of spontaneous recurrence of methamphetamine psychosis Drug Alcohol Depend 2001 64 133 142 10.1016/S0376-8716(00)00240-4 11543983

[B32] Yui K Goto K Ikemoto S Ishiguro T Stress induced spontaneous recurrence of methamphetamine psychosis: the relation between stressful experiences and sensitivity to stress Drug Alcohol Depend 2000 58 67 75 10.1016/S0376-8716(99)00060-5 10669056

[B33] Grasing K Mathur D Desouza C Written emotional expression during recovery from cocaine dependence: group and individual differences in craving intensity Subst Use Misuse 2010 45 1201 1215 10.3109/10826080903474003 20441458

[B34] Witkiewitz K Bowen S Depression, craving, and substance use following a randomized trial of mindfulness-based relapse prevention J Consult Clin Psychol 2010 78 362 374 20515211 10.1037/a0019172 PMC3280693

[B35] Spirito A Esposito-Smythers C Wolff J Uhl K Cognitive-behavioral therapy for adolescent depression and suicidality Child Adolesc Psychiatr Clin N Am 2011 20 191 204 10.1016/j.chc.2011.01.012 21440850 PMC3073681

[B36] Penn DL Mueser KT Tarrier N Gloege A Cather C Serrano D Otto MW Supportive therapy for schizophrenia: possible mechanisms and implications for adjunctive psychosocial treatments Schizophr Bull 2004 30 101 112 10.1093/oxfordjournals.schbul.a007055 15176765

[B37] Gawin FH Kleber HD Abstinence symptomatology and psychiatric diagnosis in cocaine abusers Clinical observations. Arch Gen Psychiatry 1986 43 107 113 10.1001/archpsyc.1986.01800020013003 3947206

[B38] Pickens CL Airavaara M Theberge F Fanous S Hope BT Shaham Y Neurobiology of the incubation of drug craving Trends Neurosci 2011 34 411 420 10.1016/j.tins.2011.06.001 21764143 PMC3152666

[B39] Nader MA Daunais JB Moore T Nader SH Moore RJ Smith HR Friedman DP Porrino LJ Effects of cocaine self-administration on striatal dopamine systems in rhesus monkeys: initial and chronic exposure Neuropsychopharmacology 2002 27 35 46 10.1016/S0893-133X(01)00427-4 12062905

[B40] Chang L Alicata D Ernst T Volkow N Structural and metabolic brain changes in the striatum associated with methamphetamine abuse Addiction 2007 102 Suppl 1 16 32 17493050 10.1111/j.1360-0443.2006.01782.x

[B41] Ernst T Chang L Adaptation of brain glutamate plus glutamine during abstinence from chronic methamphetamine use J Neuroimmune Pharmacol 2008 3 165 172 10.1007/s11481-008-9108-4 18521756 PMC2575014

[B42] Koob G Kreek MJ Stress, dysregulation of drug reward pathways, and the transition to drug dependence Am J Psychiatry 2007 164 1149 1159 10.1176/appi.ajp.2007.05030503 17671276 PMC2837343

[B43] Baicy K London ED Corticolimbic dysregulation and chronic methamphetamine abuse Addiction 2007 102 Suppl 1 5 15 17493049 10.1111/j.1360-0443.2006.01777.x

[B44] Chauvet C Lardeux V Goldberg SR Jaber M Solinas M Environmental enrichment reduces cocaine seeking and reinstatement induced by cues and stress but not by cocaine Neuropsychopharmacology 2009 34 2767 2778 10.1038/npp.2009.127 19741591 PMC3178884

[B45] Lynch WJ Piehl KB Acosta G Peterson AB Hemby SE Aerobic exercise attenuates reinstatement of cocaine-seeking behavior and associated neuroadaptations in the prefrontal cortex Biol Psychiatry 2010 68 774 777 10.1016/j.biopsych.2010.06.022 20692647 PMC2949528

